# Optimization of Chemical Extraction Conditions of Dietary Fiber from *Cistanche deserticola* Residues and Its Structural Characteristics and Physicochemical and Functional Properties

**DOI:** 10.3390/molecules28227604

**Published:** 2023-11-15

**Authors:** Ziming Sun, Yuanyuan Zhao, Zhen Zhang, Li Wang, Jianming Du, Shengxiang Zhang

**Affiliations:** College of Food Science and Engineering, Gansu Agricultural University, Lanzhou 730070, China; 17852850311@163.com (Z.S.); m15294207998@163.com (L.W.); my17339830698@163.com (J.D.); 18394482837@163.com (S.Z.)

**Keywords:** *Cistanche deserticola*, waste utilization, dietary fiber, response surface methodology

## Abstract

*Cistanche deserticola* residues are by-products of the industrial production of *Cistanche deserticola*, which are currently often discarded, resulting in the waste of resources. In order to achieve the efficient utilization of *Cistanche deserticola*, dietary fiber from *Cistanche deserticola* residues was extracted chemically and the optimization of the extraction conditions was performed, using the response surface methodology to study the effects of the NaOH concentration, extraction temperature, extraction time, and solid–liquid ratio on the yield of water-soluble dietary fiber (SDF). The structural, physicochemical, and functional properties of the dietary fiber were also investigated. The results showed that the optimal conditions were as follows: NaOH concentration of 3.7%, extraction temperature of 71.7 °C, extraction time of 89.5 min, and solid–liquid ratio of 1:34. The average yield of SDF was 19.56%, which was close to the predicted value of 19.66%. The two dietary fiber types had typical polysaccharide absorption peaks and typical type I cellulose crystal structures, and the surface microstructures of the two dietary fiber types were different, with the surface of SDF being looser and more porous. Both dietary fiber types had good functional properties, with SDF having the strongest water-holding capacity and the strongest adsorption capacity for nitrite, cholesterol, sodium cholate, and glucose, while IDF had a better oil-holding capacity. These results suggest that *Cistanche deserticola* residues are a good source of dietary fiber and have promising applications in the functional food processing industry.

## 1. Introduction

*Cistanche deserticola* is a traditional and valuable Chinese herbal medicine with high edible value that grows mainly in the desert areas of Northwest China [1]. *Cistanche deserticola* contains phenylethanol glycosides, polysaccharides, and flavonoids [2,3,4], giving it anti-inflammatory and antioxidant functions, improved immunity, and other pharmacological effects [5,6]. In recent years, as individuals’ concerns for their health have increased, more products derived from Cistanche cistannii have been developed, such as Cistanche cistannii wine and Cistanche cistannii beverages, and the production of Cistanche cistannii products has increased annually [7,8,9]. In industrial production, large amounts of *Cistanche deserticola* residues are generated, which are usually discarded, but these residues are rich in dietary fiber, thus representing a considerable waste of resources if discarded. Making full use of these residues can increase the added value of Cistanchis resources and improve the economic benefits.

Dietary fiber (DF) is a class of carbohydrate that is not digested and absorbed by endogenous enzymes in the human body [10]. In recent years, studies have shown that the adequate intake of dietary fiber can help to reduce the risk of obesity, diabetes mellitus, cardiovascular and cerebral vascular diseases, gastric and intestinal cancers, and other chronic diseases [11,12,13]. DF is classified into water-soluble dietary fiber (SDF) and water-insoluble dietary fiber (IDF) according to its solubility and its different physiological functions, based on its composition and structure [14]. SDF is composed of non-cellulosic polysaccharides, including β-glucan, pectin, guar gum, arabinoxylan, inulin, etc. [15]. It has the effects of lowering blood lipids, lowering blood glucose, and preventing gastric cancer, etc. [16]. IDF is mainly composed of cellulose, hemicellulose, chitosan, lignin, and other components of the plant cell wall [17], and it can increase the volume of fecal matter and accelerate the speed of intestinal transport [18]. DF is increasingly widely used in the food industry due to its unique physicochemical properties and functional properties, such as its water-holding capacity (WHC), oil-holding capacity (OHC), and other properties that contribute to its role in improving the organoleptic properties and processing characteristics of foods after it is added as an additive or nutrient fortification agent [19]. At present, dietary fiber is mainly used in foods such as pasta products, dairy products, meat products, and beverages [20].

Typically, SDF has higher value than IDF because of its better functional properties. Raw materials with SDF content comprising more than 10% of the DF can be considered high-quality sources of dietary fiber. Much of the DF currently used in the food industry has relatively low SDF content, so researchers are seeking high-quality sources of dietary fiber [21]. The chemical method of obtaining DF includes drying and crushing the raw materials, using acidic and alkaline reagents, and obtaining DF under suitable conditions [22]. The principle is to remove the proteins and fats in the raw materials using acidic and alkaline reagents so that the glycosidic bond breaks to produce a new reducing end, reducing the polymerization of fiber macromolecules, and converting them into non-digestible soluble polysaccharides. This method is suitable for large-scale application due to its simple operation and low cost [23].

Currently, there are no reports on the study of dietary fiber in Cistanchis dregs. The objective of this study is to optimize the process conditions for the chemical extraction of SDF from Cistanchis dregs using response surface optimization. By determining the structural characteristics and physicochemical and functional properties of SDF, IDF, and the raw residue of Cistanchis dregs, a theoretical basis for the utilization of Cistanchis resources and the application of dietary fiber in the food industry is provided.

## 2. Results

### 2.1. Response Surface Optimization Results

#### 2.1.1. Optimization of SDF Extraction

In this study, we chose to use the chemical method to extract SDF from *Cistanche deserticola* residues in order to optimize the extraction conditions to obtain more SDF, studying the NaOH concentration, extraction temperature, extraction time, and solid–liquid ratio as independent variables to optimize the extraction conditions. The design of the experimental factor levels is shown in Table 1.

A total of 29 sets of tests were conducted, and the results are shown in Table 2. The data in Table 2 were analyzed by multiple regression to obtain the regression equations between the SDF yield and the test factors:

Y = 19.40 − 1.28 X_1_ + 0.63 X_2_ − 0.37 X_3_ − 0.34 X_4_ − 0.57 X_1_ X_2_ − 0.42 X_1_ X_3_ − 0.13 X_1_ X_4_ + 0.55 X_2_ X_3_ − 0.35 X_2_ X_4_ − 0.25 X_3_ X_4_ − 2.55 X_1_^2^ − 2.34 X_2_^2^ − 1.12 X_3_^2^ − 1.07 X_4_^2^

The ANOVA results are shown in Table 3. The results show that the correlation coefficient of the model, R^2^ = 0.9709, was close to that of the R^2^adj of 0.9419, and both were close to 1, indicating that this model was well fitted. The *p*-value was < 0.0001, the coefficient of variation (C.V.%) of the model was 2.78% (<5%), and the out-of-fit term was 0.1445, indicating that the model could predict the SDF yield accurately. The results of the significance analysis of the coefficients of the regression model in Table 3 show that the primary coefficients X_1_, X_2_, X_3_, and X_4_; the interaction term coefficients X_1_ X_2_ and X_2_ X_3_; and the quadratic coefficients X_1_^2^, X_2_^2^, X_3_^2^, and X_4_^2^ had a significant effect on the SDF yield.

#### 2.1.2. Response Surface Analysis

**Figure 1:**
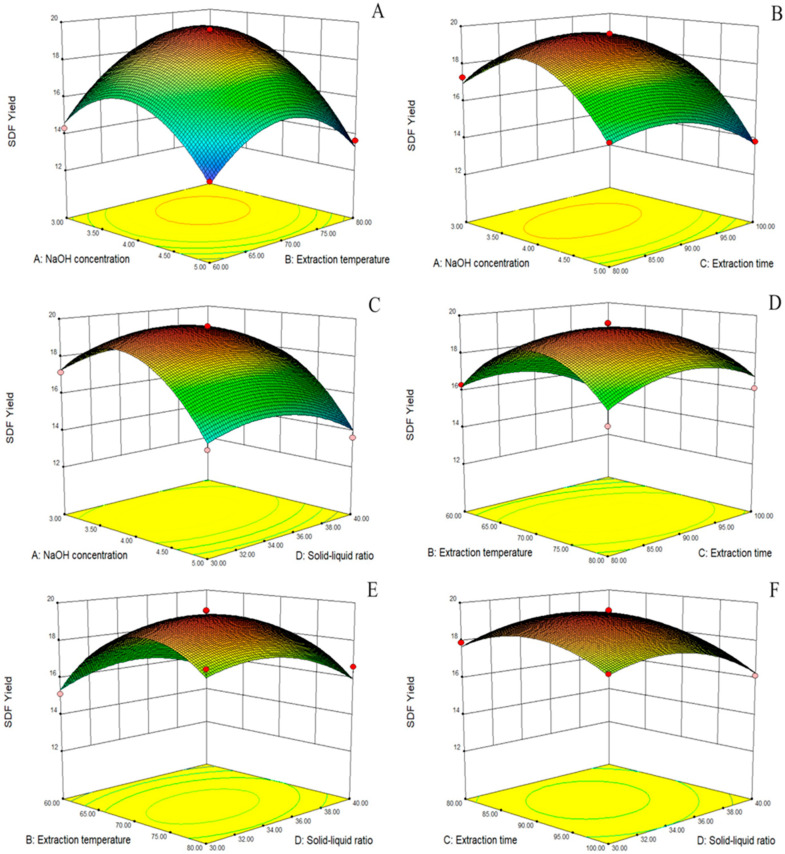
Response surface diagram. Effect of NaOH concentration and extraction temperature on SDF yield (**A**); effect of NaOH concentration and extraction time on SDF yield (**B**); effect of NaOH concentration and solid–liquid ratio on SDF yield (**C**); effect of extraction temperature and extraction time on SDF yield (**D**); effect of extraction temperature and solid–liquid ratio on SDF yield (**E**); effect of extraction time and solid–liquid ratio on SDF yield (**F**).

The 3D response surface plots generated by the model are shown in Figure 1A–F. The response surface allowed us to examine the degree of influence of each factor on the SDF yield. The different slopes of the response surfaces indicate that the interaction terms had different effects on the SDF yield of *Cistanche deserticola* residues; the greater the slope of the interaction surface, the higher the degree of its effect. The response surface and the ANOVA of the regression model showed that the order of the four factors by effect on the SDF yield was as follows: NaOH concentration > extraction temperature > extraction time > solid–liquid ratio. In addition, there was a significant correlation between the NaOH concentration and extraction temperature and a significant interaction between the extraction temperature and extraction time.

#### 2.1.3. Optimization of Parameters and Verification of the Model

According to the results of the response surface analysis, the optimal extraction conditions for SDF were as follows: NaOH concentration of 3.7%, extraction temperature of 71.7 °C, extraction time of 89.5 min, and solid–liquid ratio of 1:34. Under these optimal conditions, the maximum predicted value of the SDF yield was 19.66%. Thus, a validation experiment was conducted with these conditions and the experiment was repeated three times, resulting in an average SDF yield of 19.56%, which was not significantly different from the predicted value. This showed that the response surface optimization experiment was effective and the established regression equation model was reasonable, being suitable for predicting the extraction rate of MI-SDF.

### 2.2. SEM

**Figure 2:**
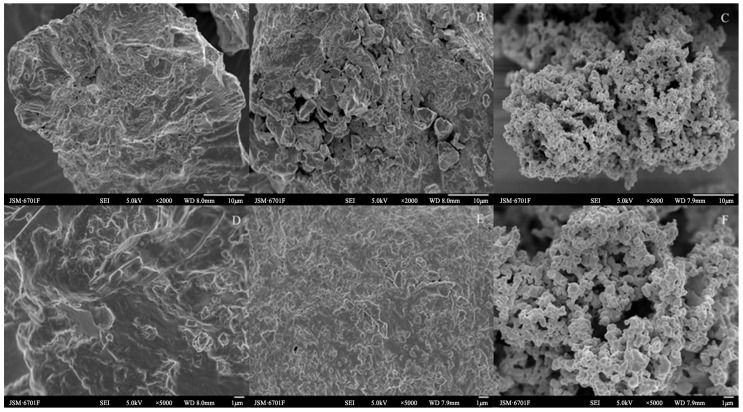
SEM images of CK (**A**,**D**), IDF (**B**,**E**), and SDF (**C**,**F**) ((**A**–**C**): ×2000; (**D**–**F**): ×5000).

As can be seen from Figure 2A–F, the surface microstructures of the *Cistanche deserticola* residues for the control check (CK), IDF, and SDF were quite different. The surface of the CK was relatively smooth compared to the IDF and SDF, and it was partially wrinkled and presented a denser structure. The folds and cracks on the surface of the IDF were increased compared to the CK, with the structure fragmented and collapsed and more small pores appearing. It also showed an increase in the ratio of the specific surface area, and the surface had particles attached, which may have been residual impurities such as protein [24]. The SDF changed from the original pleated furrow shape to a honeycomb pore-like structure formed by the agglomeration of multiple fine particles together. Further, the specific surface area and total pore volume of the particles increased, which increased their adsorption sites, and this structure gave the SDF a large specific surface area, providing it with good adsorption performance [25]. The reason for this structural change might be that the acid and alkali treatment during the extraction process broke the glycosidic bonds of the fibrous polysaccharides and decreased the polymerization of the fibrous macromolecules, which destroyed the original dense structure. This structural difference may affect the physicochemical and functional properties, such as the adsorption capacity for NO_2_^−^, cholesterol, and glucose [26].

### 2.3. FTIR

**Figure 3:**
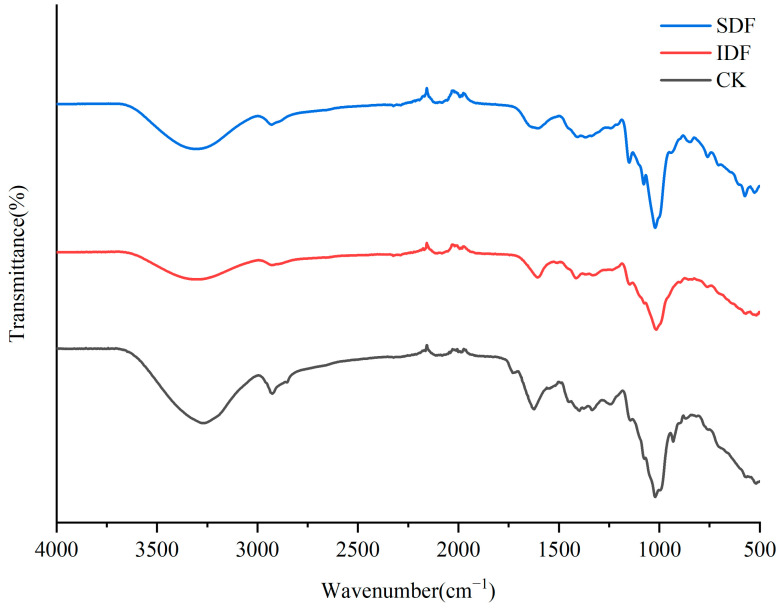
FTIR of CK, IDF, and SDF.

The FTIR profiles of the CK, IDF, and SDF are shown in Figure 3. A broad absorption peak at 3300 cm^−1^ for each sample was caused by the -O H stretching vibration of cellulose and hemicellulose. The weak absorption peak near 2929 cm^−1^ resulted from the contraction vibration of the polysaccharide methyls and methylene group C-H, which is typical of the hemicellulose structure [27]. The peak at 1609 cm^−1^ was due to the contraction vibration of C=O in the polysaccharide structure, indicating the presence of glyoxalate in the sample [28]. The absorption peak at 1394 cm^−1^ was related to the contraction vibration of O-H and the bending vibration of C-H. The strong absorption peaks near 900~1200 cm^−1^ were due to the contraction vibration of C-O, which might have been due to -C H O and C-O-C in glucose and xyloglucan. The absorption peak at 573 cm^−1^ could be attributed to the β-type C-H variable angle vibrations [29].

### 2.4. XRD

**Figure 4:**
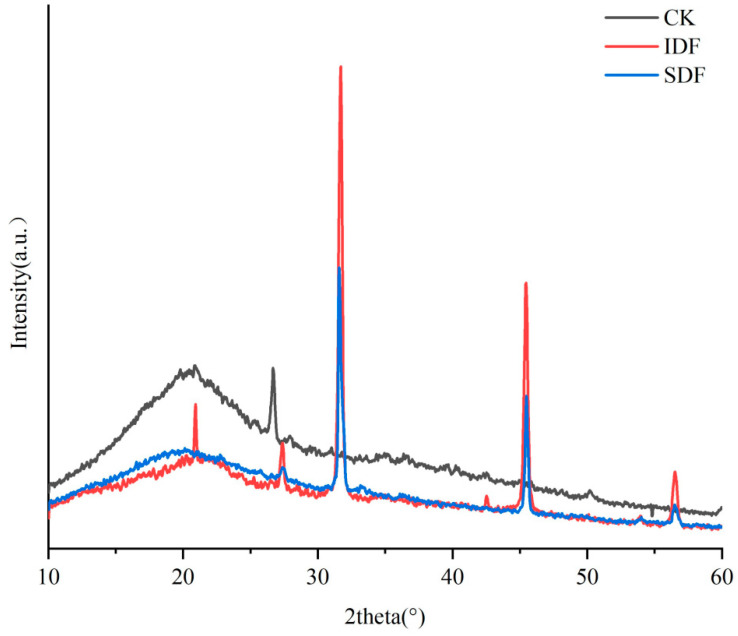
X-ray diffraction (XRD) patterns of CK, IDF, and SDF.

XRD is an analytical method that reflects the crystalline properties and crystallinity of a substance through crystal diffraction phenomena. The crystallinity of DF directly affects its physical and chemical properties, such as water retention, oil retention, and swelling. The X-ray diffraction patterns of the CK, IDF, and SDF are shown in Figure 4. All three exhibited a gentle and broad crystalline peak near 2θ = 20°, which indicates that they had a predominantly amorphous structure in the crystalline region, which is typical of type I cellulose [30]. They displayed the typical crystal structure of type I cellulose. At 2θ = 27°, 31°, and 45°, the peak intensity of the IDF was significantly higher than that of the SDF, indicating that the crystal structure of the IDF was more ordered and the crystallinity was higher than that of the SDF [31]. The low crystallinity of the SDF indicated that the original crystalline region of the molecule had been destroyed, the degree of polymerization had been reduced, and the surface structure was looser, which explained the high adsorption capacity of the SDF from the side [32].

### 2.5. Composition of Monosaccharides

The monosaccharide compositions of the SDF and the IDF from *Cistanche deserticola* residues are shown in Table 4, with seven monosaccharides detected in SDF and eight in IDF. The most prominent monosaccharide in both dietary fiber types was glucose, which is mainly derived from cellulose in the cell wall. The glucose content of the IDF was higher than that of the SDF, which could be attributed to the alkaline conditions that promoted the hydrolysis of cellulose in the cell wall to glucose, as well as glucose oligosaccharides and dextrins, which were composed of glucose units. Meanwhile, the SDF contained more arabinose, galactose, and rhamnose than the IDF, which suggested the presence of pectin substances and soluble polysaccharides produced by the hydrolysis of cellulose or hemicellulose in the SDF. Some studies have shown that polysaccharides containing arabinose and rhamnose could lower blood glucose levels and regulate lipid metabolism and other physiological activities, so SDF may have better hypoglycemic and hypolipidemic properties than IDF [33,34].

### 2.6. Physicochemical Properties

#### WHC and OHC

The WHC and OHC of the CK, IDF, and SDF are shown in Table 5. The high water-holding capacity of DF allows it to be added to food as a functional ingredient to prevent dehydration and change the viscosity, for example. The results showed that the WHC of the SDF was slightly higher than that of the IDF, which was probably due to the fact that the surface of the SDF was looser, which was favorable for the infiltration of more water. In addition, the WHC was related to the amount of hydrophilic groups exposed—the more hydrophilic groups that were exposed, the higher the water absorption was [34]. Meanwhile, the OHC of the IDF was higher than that of the SDF. This was because the OHC of DF is positively correlated with the lignin content, which was higher in the IDF. In addition, the surface characteristics, the total charge density, and the hydrophobicity also increased the OHC of the IDF [35]. A good OHC helps to maintain the stability of high-fat food products, and it has a positive effect by enhancing the product texture [34].

### 2.7. Functional Properties

#### 2.7.1. NIAC

**Figure 5:**
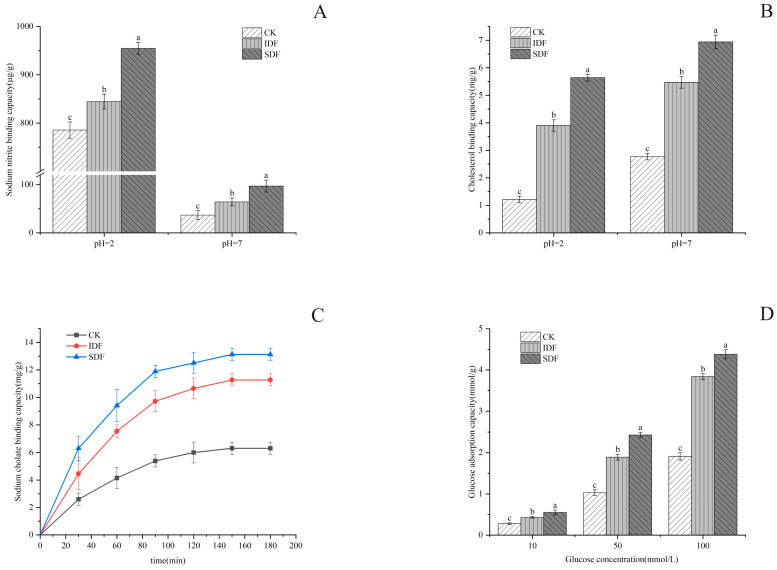
NIAC (**A**), CAC (**B**), SAC (**C**), and GAC (**D**) for CK, IDF, and SDF. The different letters indicate significant differences within groups ((**A**,**B**,**D**), *p* < 0.05).

Nitrite was chemically reacted with secondary amines and amides to form carcinogens (*N*-nitroso compounds) under acidic conditions. The results of this study are shown in Figure 5A, according to which the adsorption amount of all samples at pH = 2 was much larger than that at pH = 7. This was because, under acidic conditions, NO_2_^−^ could combine with H^+^ to produce nitrogen oxides such as HNO_2_ and N_2_O_3_, and these nitrogen oxides could combine with the negatively charged oxygen atoms in the phenolic acid groups of the dietary fibers to undergo chemisorption [36]. The highest adsorption under the simulated stomach conditions was found in SDF (970.58 μg/g), followed by IDF (861.55 μg/g), and the lowest was found in the CK (805.99 μg/g) due to its structure. The sparser the structure of the sample is, the larger the specific surface area is, and more binding sites and adsorption sites lead to the better adsorption of NO_2_^−^ [36].

#### 2.7.2. CAC

In this experiment, the adsorption effect of the CK, IDF, and SDF on cholesterol in a gastrointestinal environment was simulated by measuring the decrease in yolk cholesterol content under different pH conditions. As shown in Figure 5B, the three samples all reduced the content of cholesterol in the egg yolk to varying degrees, and the adsorption effect was different under different pH conditions. The adsorption effect at pH = 7 was stronger than that at pH = 2, indicating that the adsorption of cholesterol in the small intestine environment is better than that in gastric juice [37]. This is due to the presence of a large amount of H^+^ in the acidic environment, and DF and cholesterol themselves have positively charged groups, which would have a repulsive effect and reduce the adsorption of cholesterol [36]. Under the condition of pH = 7, SDF had the strongest ability to adsorb cholesterol (9.63 mg/g), followed by IDF (8.07 mg/g) and CK (4.16 mg/g). Under the condition of pH = 2, SDF had the best adsorption effect on cholesterol (2.6 mg/g), followed by IDF (1.82 mg/g) and CK (1.3 mg/g).

#### 2.7.3. SAC

Bile acids are normally stored in the gallbladder and enter the small intestine after a person has eaten, to participate in hepatic and intestinal circulation and regulate the metabolism of cholesterol in the human body. Most bile acids are in the form of sodium cholate, and the ability of a sample to bind with sodium cholate is also an important indicator in evaluating its cholesterol-lowering ability [38]. As shown in Figure 5C, the sodium cholate adsorption level of all samples increased gradually with time and began to stabilize after 120 min, but the adsorption capacity of SDF was much larger than that of IDF and CK because of its large specific surface area. Some other studies have shown that an increase in the ability to form a gel would enhance the ability to adsorb sodium cholate, and this may also be the reason for the higher adsorption capacity of SDF [39].

#### 2.7.4. GAC

The adsorption of glucose by DF can reduce the absorption of glucose by the human body to a certain extent, which is important in reducing the elevation in blood glucose after meals [40]. As shown in Figure 5D, the CK, IDF, and SDF adsorbed glucose in the solution to a certain extent, and this was positively correlated with the concentration of glucose. The GAC of the SDF was the highest, reaching 4.446 mmol/g when the concentration of glucose was 100 mmol/L. The GAC of the IDF was slightly lower than that of the SDF, and the GAC of the CK was significantly lower than that of the SDF and IDF. The reason that the GAC of the SDF was higher than that of the IDF and CK was due to its loose surface, with a larger surface area and more pores, which facilitated the entry of molecules and the adsorption and retention of glucose [24].

## 3. Materials and Methods

### 3.1. Reagents and Chemicals

*Cistanche deserticola* residues were provided by Gansu Rudan Pharmaceutical Co., Ltd. (Lanzhou, China). The monosaccharide standards were HPLC-grade. HCL, H_2_SO_4_, and cholesterol were purchased from Sinopharm Chemical Reagent Co., Ltd. (Shanghai, China). Sodium cholate and o-phthalaldehyde were purchased from Shanghai Yuanye Bio-Technology Co., Ltd. (Shanghai, China). NaOH and sodium nitrite were purchased from Tianjin Baishi Chemical industry Co., Ltd. (Tianjin, China). Furfural was purchased from Tianjin Damao Chemical Reagent Factory (Tianjin, China). Anhydrous glucose and *N*-1-naphthylethylenediamine dihydrochloride were purchased from Tianjin Guangfu Fine Chemical Research Institute (Tianjin, China). Acetic acid and anhydrous ethanol were purchased from Chengdu Chron Chemicals Co., Ltd. (Chengdu, China). 4-Aminobenzenesulfonic acid was purchased from Yantai Shuangshuang Chemical Co., Ltd. (Yantai, China). DNS reagent was purchased from Phygene Biotechnology Co., Ltd. (Fuzhou, China). 

### 3.2. Extraction of SDF and IDF

#### 3.2.1. Extraction of Dietary Fiber

Dietary fiber was extracted according to the method of Wang et al. [35] with slight modifications. Five grams of powder was mixed with NaOH at a certain solid–liquid ratio, and the water bath was heated for a certain time. Then, the extract was centrifuged (6000 RPM, 15 min) to separate the precipitate from the supernatant, and the precipitate was washed with distilled water and anhydrous ethanol in turn until neutral and placed in an oven for drying to obtain the IDF. To obtain the SDF, 10% HCl was added to the supernatant, the pH was adjusted to 3.8, and it was placed into a refrigerated environment at 4 °C to precipitate for 12 h. Then, the extract was centrifuged to collect the supernatant, four times the volume of anhydrous ethanol (95%) was added to the supernatant, and it was left to stand at room temperature for 12 h. After this, the mixture was centrifuged to obtain the precipitate, which was washed with anhydrous ethanol repeatedly until neutral and then placed into an oven (40 °C) to dry.

$$\mathrm{The}\;\mathrm{yield}\;\mathrm{of}\;\mathrm{dietary}\;\mathrm{fiber}\;\left(\%\right)=\frac{\mathrm{Weight}\;\mathrm{of}\;\mathrm{extracted}\;\mathrm{fiber}}{\mathrm{Sample}\;\mathrm{weight}}\times 100$$

#### 3.2.2. Optimization of Extraction Conditions

Based on the previous one-factor test, the NaOH concentration, extraction temperature, extraction time, and solid–liquid ratio were determined as independent variables, recorded as X_1_, X_2_, X_3_, and X_4_, respectively. A response surface test was carried out by using the Box–Behnken central combination experimental design, with the SDF yield as the response value, and the design of the experimental factor levels is shown in Table 1. The results of the response surface analysis with the Box–Behnken test were analyzed using the Design-Expert 8.0.6 software, and the test results are shown in Table 2.

### 3.3. Scanning Electron Microscopy (SEM)

The ground and sieved sample powder was placed on a carrier stage with a conductive adhesive. The excess sample was carefully blown off with a wash ball and then removed after the surface was gold-plated using ion sputtering coating, and the sample was then placed under a scanning electron microscope (JSM-6701F, JEOL, JPN) for scanning observation.

### 3.4. Fourier Transform Infrared Spectrometry (FTIR)

Referring to Ma et al. [41], the dried samples were separately mixed with dried potassium bromide powder in a mortar, ground through a sieve, and pressed into thin slices, and the Nicolet™ iS50 FTIR spectrometer (Nicolet iS50u, Thermo Fisher Scientific, Madison, WI, USA) was used to scan for near-infrared spectra between 400 and 4000 cm^−1^.

### 3.5. X-ray Diffraction (XRD)

Referring to Song et al. [42], the samples were placed into a specialized sample tank, compacted with a glass plate, and then transferred to a diffractometer (XD-3, Beijing General Analytical Instrument Co., Ltd., Beijing, China) to analyze the crystal structure of the samples by X-ray diffraction analysis. The voltage of the instrument was adjusted to 40 kV and the current to 40 mA, and the target type of Cu-Kα was used with an angular step of 0.02° and a scanning range of 10–60°.

### 3.6. Composition of Monosaccharides

The sample extracts were analyzed by high-performance anion-exchange chromatography (HPAEC) on a CarboPac PA-20 anion-exchange column (3 by 150 mm; Dionex) using a pulsed amperometric detector (PAD; Dionex ICS 5000+ system, Thermo Fisher Scientific, USA). The settings were as follows: flow rate, 0.5 mL/min; injection volume, 5 µL; solvent system A, ddH_2_O; solvent system B, 0.1 M NaOH; solvent system C, 0.1 M NaOH, 0.2 M NaAc. The gradient program was as follows: volume ratio of solutions A, B, and C was 95:5:0 at 0 min, 85:5:10 at 26 min, 85:5:10 at 42 min, 60:0:40 at 42.1 min, 60:40:0 at 52 min, 95:5:0 at 52.1 min, and 95:5:0 at 60 min [43]. The standard monosaccharides, including fucose, rhamnose, arabinose, galactose, glucose, xylose, mannose, galacturonic acid, and glucuronic acid, were analyzed in the hydrolyzed samples.

### 3.7. Physicochemical Properties

#### 3.7.1. Water-Holding Capacity (WHC)

The WHC was determined referring to the method of Yang et al. [44], with slight modifications. Initially, 1.00 g (m_1_) of the sample was accurately weighed, to which 10 mL of water was added; this was then mixed well and left at a constant temperature of 37 °C for 24 h. The sample was then freeze-centrifuged at 4800 rpm for 10 min, the residue was immediately extracted, and the weight was measured (m_2_). Finally, the WHC of the sample was calculated according to the following equation:

$$\mathrm{WHC}(g/g)=\frac{m_{2}-m_{1}}{m_{1}}$$

where m_1_ and m_2_ are the mass of the sample before and after adsorption, respectively.

#### 3.7.2. Oil-Holding Capacity (OHC)

The OHC was determined referring to the method of Zhang et al. [45], with slight modifications. Here, 1.00 g of sample (m_1_) was weighed and placed in a centrifuge tube, to which 25 mL of soybean oil was added. This was then placed at a constant temperature of 37 °C for 2 h, the precipitate was taken after centrifugation at 4800 r/min for 20 min, and the weight (m_2_) was determined immediately. The OHC of the sample was calculated according to the following equation:

$$\mathrm{OHC}(g/g)=\frac{m_{2}-m_{1}}{m_{1}}$$

where m_1_ and m_2_ are the mass of the sample before and after adsorption, respectively.

### 3.8. Functional Properties

#### 3.8.1. Nitrite Ion Adsorption Capacity (NIAC)

The NIAC was determined referring to the method of Luo et al. [46], with slight modifications. Briefly, 0.1 g of dried sample was added to 5 mL of 20 μg/mL NaNO_2_ solution, and the pH was adjusted to 7.0 and 2.0 to simulate the small intestine and stomach environments, respectively. Then, it was allowed to stand at room temperature for 2 h. The sample was centrifuged at 4800 rpm for 10 min and then centrifuged again to remove 0.4 mL of the supernatant in 10 mL colorimetric tubes. The content of sodium nitrite in the supernatant was determined using *N*-(1-naphthyl)-ethylenediamine dihydrochloride spectrophotometry. The standard curve was plotted using standard NaNO_2_ solution. The NIAC of the sample was calculated according to the following equation:

$$\mathrm{NIAC}(\mu g/g)=\frac{\left(C_{1}-C_{2}\right)V}{W}$$

where C_1_ and C_2_ are the concentration of NaNO_2_ in the supernatant before and after adsorption, respectively; W is the mass of the sample; and V is the volume of NaNO_2_ solution.

#### 3.8.2. Cholesterol Adsorption Capacity (CAC) 

The CAC was determined with reference to the method of Deng et al., with minor modifications [47]. Fresh eggs were taken, and the yolks were mixed well with ultrapure water at 1:9 to determine the mass of cholesterol in the diluted yolk solution using the o-phthalaldehyde method, noted as W_1_. Here, 1 g of the sample and 40 mL of the yolk dilution were placed in a centrifuge tube, mixed well, adjusted to pH = 2.0 and pH = 7.0 to simulate the gastrointestinal environment, shaken at 37 °C for 4 h, and then centrifuged for 15 min using a refrigerated centrifuge. The mass of cholesterol was determined and recorded as W_2_. The CAC of the sample was calculated according to the following equation:

$$\mathrm{CAC}(\mathrm{mg}/g)=\frac{W_{1}-W_{2}}{m}$$

where W_1_ and W_2_ are the mass of cholesterol before and after adsorption, respectively, and m is the mass of the sample.

#### 3.8.3. Sodium Cholate Adsorption Capacity (SAC)

The SAC was determined with reference to the method of Benitez et al., with minor modifications [48]. First, 0.25 g of sample and 0.05 g of sodium cholate were taken and mixed with 25 mL of NaCl (0.15 mol/L) solution and then shaken in the middle at 37 ℃. Next, the appropriate amount of solution was removed from the system at 30, 60, 90, 120, 150, and 180 min and centrifuged for 10 min, and the content of sodium cholate in the supernatant was determined by the furfural colorimetric method. The standard curve was plotted using standard sodium cholate solution. The SAC of the sample was calculated according to the following equation:

$$\mathrm{SAC}(\mathrm{mg}/g)=\frac{W_{1}-W_{2}}{m}$$

where W_1_ and W_2_ are the mass of sodium cholate before and after adsorption, respectively, and m is the mass of the sample.

#### 3.8.4. Glucose Adsorption Capacity (GAC)

The GAC was determined with reference to the method of Ma et al., with minor modifications [49]. First, 100 mg of the sample was accurately weighed and poured into a centrifuge tube. Next, 10 mL of glucose solution (10, 50, 100 mmol/L) was taken and transferred into the above centrifuge tube, which was oscillated at 37 °C for 6 h and then centrifuged in a high-speed freezer centrifuge. Lastly, the glucose content in the supernatant was determined by the DNS method. The standard curve was plotted using standard glucose solution. The GAC of the sample was calculated according to the following equation:

$$\mathrm{GAC}(\mu \mathrm{mol}/g)=\frac{(C_{1}-C_{2})V}{m}$$

where C_1_ and C_2_ are the glucose content before and after adsorption, respectively; V is the supernatant volume; and m is the sample mass.

### 3.9. Statistical Analysis

All experiments were repeated 3 times, and the data were processed by SPSS 25.0. The final experimental results are expressed as the mean ± standard deviation, and *p* < 0.05 was considered significant.

## 4. Conclusions

In this study, dietary fiber was extracted from *Cistanche deserticola* residues using the chemical method, and the response surface methodology was used to optimize the yield of SDF. The optimal conditions were as follows: NaOH concentration of 3.7%, extraction temperature of 71.7 °C, extraction time of 89.5 min, and solid–liquid ratio of 1:34. The average yield of SDF was 19.56%. The structure and adsorption properties of the dietary fiber were also investigated. Both dietary fiber types studied had typical polysaccharide absorption peaks and a typical cellulose crystal structure of type I. The surface microstructures of the SDF and IDF were different, with the surface of the SDF being looser and more porous. The two dietary fiber types had different monosaccharide compositions, with glucose being the main component in both. The SDF’s water-holding capacity, the adsorption of nitrite and cholesterol under acidic conditions, and the adsorption of sodium cholate and glucose were stronger than those of the CK and IDF. The oil-holding capacity of the IDF was the highest. In summary, the dietary fiber in *Cistanche deserticola* residues has good water-holding capacity, oil-holding capacity, and adsorption capacity, and it has application prospects in the functional food processing industry. On this basis, functional foods with hypolipidemic and hypoglycemic effects could be developed, such as chewable tablets.

## Figures and Tables

**Table 1:** Box–Behnken design of test factor levels and coding.

Level	X_1_ NaOH Concentration (%)	X_2_ Extraction Temperature (°C)	X_3_ Extraction Time (min)	X_4_ Solid–Liquid Ratio
−1	3	60	80	1:30
0	4	70	90	1:35
1	5	80	100	1:40

**Table 2:** Box–Behnken design test results.

Run	X_1_	X_2_	X_3_	X_4_	Yield (%)
1	4	70	90	35	19.57
2	4	70	100	40	16.12
3	3	80	90	35	16.94
4	4	70	80	30	17.89
5	5	70	90	30	14.65
6	4	80	80	35	15.63
7	4	70	90	35	19.27
8	4	60	80	35	16.31
9	4	70	90	35	18.95
10	4	80	90	30	17.71
11	3	70	90	30	17.15
12	5	70	80	35	15.36
13	3	70	90	40	16.62
14	4	60	90	30	15.12
15	4	80	90	40	16.61
16	4	60	90	40	15.43
17	3	60	90	35	14.31
18	3	70	80	35	17.28
19	4	70	90	35	19.6
20	4	80	100	35	16.13
21	3	70	100	35	17.41
22	4	60	100	35	14.61
23	4	70	90	35	19.62
24	5	80	90	35	13.65
25	5	70	100	35	13.82
26	5	70	90	40	13.62
27	4	70	80	40	17.51
28	5	60	90	35	13.31
29	4	70	100	30	17.48

**Table 3:** Response surface test ANOVA.

Source	Sum of Squares	Df	Mean Square	F-Value	*p*-Value	Significance
Model	97.93	14	7.00	33.40	<0.0001	**
A-X_1_	19.51	1	19.51	93.13	<0.0001	**
B-X_2_	4.79	1	4.79	22.86	0.0003	**
C-X_3_	1.62	1	1.62	7.74	0.0147	*
D-X_4_	1.39	1	1.39	6.66	0.0218	*
AB	1.31	1	1.31	6.26	0.0254	*
AC	0.70	1	0.70	3.33	0.0895	
AD	0.063	1	0.063	0.30	0.5935	
BC	1.21	1	1.21	5.78	0.0307	*
BD	0.50	1	0.50	2.37	0.1458	
CD	0.24	1	0.24	1.15	0.3024	
A^2^	42.09	1	42.09	200.93	<0.0001	
B^2^	35.59	1	35.59	169.89	<0.0001	
C^2^	8.11	1	8.11	38.74	<0.0001	
D^2^	7.48	1	7.48	35.69	<0.0001	
Residual	2.93	14	0.21			
Lack of Fit	2.60	10	0.26	3.08	0.1445	Not significant
Pure Error	0.34	4	0.084			
Cor Total	100.87	28				
R-Squared	0.9709					
Adj R-Squared	0.9419					
Adeq Precision	18.908					
C.V. %	2.78					

* Significant at *p* < 0.05. ** Significant at *p* < 0.01.

**Table 4:** The monosaccharide compositions of IDF and SDF.

Monosaccharide Composition%	IDF	SDF
Fucose, Fuc	0.30%	0.00%
Rhamnose, Rha	1.22%	3.83%
Arabinose, Ara	9.61%	23.02%
Galactose, Gal	3.97%	7.03%
Glucose, Glc	68.47%	63.28%
Xylose, Xyl	6.80%	1.10%
Mannose, Man	1.52%	0.00%
Galacturonic Acid, Gal-UA	8.10%	1.20%
Glucuronic Acid, Glc-UA	0.00%	0.53%

**Table 5:** Physicochemical properties of CK, IDF, and SDF.

	WHC (g/g)	OHC (g/g)
CK	3.68 ± 0.07 ^a^	2.68 ± 0.11 ^c^
IDF	1.78 ± 0.09 ^c^	4.84 ± 0.08 ^a^
SDF	2.56 ± 0.12 ^b^	3.26 ± 0.09 ^b^

Different letters (^a, b, c^) in the same column indicate significantly different means at *p* < 0.05.

## Data Availability

The data presented in this study are available within the article.

## References

[1] Cheng N., Wang H., Hao H.-F., Rahman F.U., Zhang Y.M. (2023). Research progress on polysaccharide components of *Cistanche deserticola* as potential pharmaceutical agents. Eur. J. Med. Chem..

[2] Li Y.-L., Zhang Y.-L., Su X.-M., Zou P.-F., Wang X.-Y., Chen J., Zhu X. (2022). Effects of Two Kinds of Extracts of *Cistanche deserticola* on Intestinal Microbiota and Its Metabolis. Foods.

[3] Ai Z.P., Xie Y.K., Li X.Y., Lei D.W., Ambrose K., Liu Y.H. (2022). Revealing color change and drying mechanisms of pulsed vacuum steamed *Cistanche deserticola* through bioactive components, microstructural and starch gelatinization properties. Food Res. Int..

[4] Liu X.J., Wu X.L., Wang S.Y., Zhao Z.Y., Jian C., Li M.Y., Qin X.M. (2023). Microbiome and metabolome integrally reveal the anti-depression effects of *Cistanche deserticola* polysaccharides from the perspective of gut homeostasis. Int. J. Biol. Macromol..

[5] Morikawa T., Xie H.H., Pan Y.N., Ninomiya K., Yuan D., Jia X.G., Yoshikawa M., Nakamura S., Matsuda H., Muraoka O. (2019). A Review of Biologically Active Natural Products from a Desert Plant Cistanche tubulosa. Chem. Pharm. Bull..

[6] Ai Z.P., Xie Y.K., Li X.Y., Zhu G.F., Peng Z.K., Liu Y.H., Mowafy S., Guo J.L. (2023). Mechanism of freezing treatment to improve the efficiency of hot air impingement drying of steamed *Cistanche deserticola* and the quality attribute of the dried product. Ind. Crop. Prod..

[7] Lei H.B., Wang X.Y., Zhang Y.H., Cheng T.F., Mi R., Xu X.K., Zu X.P., Zhang W.D. (2020). Herba Cistanche (Rou Cong Rong): A Review of Its Phytochemistry and Pharmacology. Chem. Pharm. Bull..

[8] Zhou S.Q., Feng D., Zhou Y.X., Duan H., Jiang Y.J., Yan W.J. (2023). Analysis of the active ingredients and health applications of cistanche. Front. Nutr..

[9] Song Y.L., Zeng K.W., Jiang Y., Tu P.F. (2021). Cistanches Herba, from an endangered species to a big brand of Chinese medicine. Med. Res. Rev..

[10] Ul Ain H.B., Saeed F., Arshad M.U., Ahmad N., Nasir M.A., Amir R.M., Kausar R., Niaz B. (2018). Modification of barley dietary fiber through chemical treatments in combination with thermal treatment to improve its bioactive properties. Int. J. Food Prop..

[11] Fuller S., Beck E., Salman H., Tapsell L. (2016). New Horizons for the Study of Dietary Fiber and Health: A Review. Plant Foods Hum. Nutr..

[12] Ye S.X., Shah B.R., Li J., Liang H.S., Zhan F.C., Geng F., Li B. (2022). A critical review on interplay between dietary fibers and gut microbiota. Trends Food Sci. Technol..

[13] Tan Z.B., Meng Y., Li L., Wu Y.R., Liu C., Dong W.G., Chen C.Z. (2023). Association of Dietary Fiber, Composite Dietary Antioxidant Index and Risk of Death in Tumor Survivors: National Health and Nutrition Examination Survey 2001–2018. Nutrients.

[14] Song Y., Su W., Mu Y.C. (2018). Modification of bamboo shoot dietary fiber by extrusion-cellulase technology and its properties. Int. J. Food Prop..

[15] Zhu Y., He C.H., Fan H.X., Lu Z.X., Lu F.X., Zhao H.Z. (2019). Modification of foxtail millet (*Setaria italica*) bran dietary fiber by xylanase-catalyzed hydrolysis improves its cholesterol-binding capacity. LWT-Food Sci. Technol..

[16] Ul Ain H.B., Saeed F., Ahmed A., Khan M.A., Niaz B., Tufail T. (2019). Improving the physicochemical properties of partially enhanced soluble dietary fiber through innovative techniques:a coherent review. J. Food Process. Pres..

[17] Bashir S., Ahmad A., Abbasi K.S., Akram Z. (2022). Optimization of ultrasonic-assisted extraction of insoluble dietary fiber from wheat bran and its characterization. J. Food Process. Pres..

[18] Grosse C.S., Cope V.C. (2019). Dietary Fibre Intake and Bowel Habits After Bariatric Surgery: A Structured Literature Review. Obes. Surg..

[19] Zhang S.S., Duan J.Y., Zhang T.T., Lv M., Gao X.G. (2023). Effect of compound dietary fiber of soybean hulls on the gel properties of myofibrillar protein and its mechanism in recombinant meat products. Front. Nutr..

[20] Vilcapoma W., de Bruijn J., Elias-Penafiel C., Espinoza C., Farfan-Rodriguez L., Lopez J., Encina-Zelada C.R. (2023). Optimization of Ultrasound-Assisted Extraction of Dietary Fiber from Yellow Dragon Fruit Peels and Its Application in Low-Fat Alpaca-Based Sausages. Foods.

[21] Dong W.J., Wang D.D., Hu R.S., Long Y.Z., Lv L.H. (2020). Chemical composition, structural and functional properties of soluble dietary fiber obtained from coffee peel using different extraction methods. Food Res. Int..

[22] Xia Y.J., Meng P., Liu S.D., Tan Z.M., Yang X., Liang L.H., Xie F., Zhang H., Wang G.Q., Xiong Z.Q. (2022). Structural and Potential Functional Properties of Alkali-Extracted Dietary Fiber From Antrodia camphorate. Front. Microbiol..

[23] Jiang G.H., Ramachandraiah K., Wu Z.G., Ameer K. (2022). The Influence of Different Extraction Methods on the Structure, Rheological, Thermal and Functional Properties of Soluble Dietary Fiber from Sanchi (Panax notoginseng) Flower. Foods.

[24] Wen Y., Niu M., Zhang B., Zhao S., Xiong S. (2017). Structural characteristics and functional properties of rice bran dietary fiber modified by enzymatic and enzyme-micronization treatments. LWT-Food Sci. Technol..

[25] Zheng Y.F., Wang Q., Huang J.Q., Fang D.Y., Zhuang W.J., Luo X.L., Zou X.B., Zheng B.D., Cao H. (2019). Hypoglycemic effect of dietary fibers from bamboo shoot shell: An in vitro and in vivo study. Food Chem. Toxicol..

[26] Chen H., Xiong M., Bai T.M., Chen D.W., Zhang Q., Lin D.R., Liu Y.T., Liu A.P., Huang Z.Q., Qin W. (2021). Comparative study on the structure, physicochemical, and functional properties of dietary fiber extracts from quinoa and wheat. LWT-Food Sci. Technol..

[27] Tang Z.J., Huang G.L., Huang H.L. (2023). Ultrasonic/cellulase-assisted extraction of polysaccharide from Garcinia mangostana rinds and its carboxymethylated derivative. Ultrason. Sonochemistry.

[28] Lin B.B., Fan Y.M., Huang G.L. (2023). Preparation, analysis and properties of shaddock ped polysaccharide and its derivatives. Carbohydr. Res..

[29] Huang H.R., Chen J.J., Chen Y., Xie J.H., Liu S., Sun N., Hu X.B., Yu Q. (2021). Modification of tea residue dietary fiber by high-temperature cooking assisted enzymatic method: Structural, physicochemical and functional properties. LWT-Food Sci. Technol..

[30] Dong Y.F., Li Q., Zhao Y.H., Cao J.X. (2023). Effects of ultrasonic assisted high-temperature cooking method on the physicochemical structure characteristics and in vitro antioxidant capacities of dietary fiber from Dendrocalamus brandisii Munro shoots. Ultrason. Sonochemistry.

[31] Kaur B., Panesar P.S., Thakur A. (2021). Extraction and evaluation of structural and physicochemical properties of dietary fiber concentrate from mango peels by using green approach. Biomass Convers. Biorefinery.

[32] Sang J.Q., Li L., Wen J., Gu Q.Q., Wu J.J., Yu Y.S., Xu Y.J., Fu M.Q., Lin X. (2021). Evaluation of the Structural, Physicochemical and Functional Properties of Dietary Fiber Extracted from Newhall Navel Orange By-Products. Foods.

[33] Zhang W.M., Zeng G.L., Pan Y.G., Chen W.X., Huang W.Y., Chen H.M., Li Y.S. (2017). Properties of Soluble Dietary Fiber-Polysaccharide from Papaya Peel Obtained Through Alkaline or Ultrasound-Assisted Alkaline Extraction. Carbohydr. Polym..

[34] Jia M.Y., Chen J.J., Liu X.Z., Xie M.Y., Nie S.P., Chen Y., Xie J.H., Yu Q. (2019). Structural characteristics and functional properties of soluble dietary fiber from defatted rice bran obtained through Trichoderma viride fermentation. Food Hydrocoll..

[35] Wang K.L., Li M., Wang Y.X., Liu Z.H., Ni Y.Y. (2020). Effects of extraction methods on the structural characteristics and functional properties of dietary fiber extracted from kiwifruit (*Actinidia deliciosa*). Food Hydrocoll..

[36] Chu J.X., Zhao H.Z., Lu Z.X., Lu F.X., Bie X.M., Zhang C. (2019). Improved physicochemical and functional properties of dietary fiber from millet bran fermented by Bacillus natto. Food Chem..

[37] Lin Y.A., Chen K., Tu D., Yu X.N., Dai Z.Y., Shen Q. (2018). Characterization of dietary fiber from wheat bran (*Triticum aestivum* L.) and its effect on the digestion of surimi protein. LWT-Food Sci. Technol..

[38] Dong J.L., Wang L., Lu J., Zhu Y.Y., Shen R.L. (2019). Structural, antioxidant and adsorption properties of dietary fiber from foxtail millet (Setaria italica) bran. J. Sci. Food Agric..

[39] Zhang S.S., Xu X.L., Cao X., Liu T.T. (2022). The structural characteristics of dietary fibers from Tremella fuciformis and their hypolipidemic effects in mice. Food Sci. Hum. Wellness.

[40] Ding Q.Z., Li Z.K., Wu W., Su Y.Y., Sun N.Z., Luo L., Ma H.L., He R.H. (2020). Physicochemical and functional properties of dietary fiber from Nannochloropsis oceanica: A comparison of alkaline and ultrasonic-assisted alkaline extractions. LWT-Food Sci. Technol..

[41] Ma R., Chen J.N., Zhou X.J., Lin H., Gao Q., Peng X., Tanokura M., Xue Y.L. (2021). Effect of chemical and enzymatic modifications on the structural and physicochemical properties of dietary fiber from purple turnip (*Brassica rapa* L.). LWT-Food Sci. Technol..

[42] Song L.W., Qi J.R., Liao J.S., Yang X.Q. (2021). Enzymatic and enzyme-physical modification of citrus fiber by xylanase and planetary ball milling treatment. Food Hydrocoll..

[43] Zhang H., Li C.C., Lai P.F.H., Chen J.S., Xie F., Xia Y.J., Ai L.Z. (2021). Fractionation, chemical characterization and immunostimulatory activity of β-glucan and galactoglucan from Russula vinosa Lindblad. Carbohydr. Polym..

[44] Yang X., Dai J., Zhong Y., Wei X.L., Wu M.X., Zhang Y.X., Huang A., Wang L.J., Huang Y.K., Zhang C.S. (2021). Characterization of insoluble dietary fiber from three food sources and their potential hypoglycemic and hypolipidemic effects. Food Funct..

[45] Zhang Y., Qi J.R., Zeng W.Q., Huang Y.X., Yang X.Q. (2020). Properties of dietary fiber from citrus obtained through alkaline hydrogen peroxide treatment and homogenization treatment. Food Chem..

[46] Luo X.L., Wang Q., Fang D.Y., Zhuang W.J., Chen C.H., Jiang W.T., Zheng Y.F. (2018). Modification of insoluble dietary fibers from bamboo shoot shell: Structural characterization and functional properties. Int. J. Biol. Macromol..

[47] Deng M., Lin Y.S., Dong L.H., Jia X.C., Shen Y.L., Liu L., Chi J.W., Huang F., Zhang M.W., Zhang R.F. (2021). Physicochemical and functional properties of dietary fiber from pummelo (*Citrus grandis* L. Osbeck) and grapefruit (*Citrus paradisi* Mcfad) cultivars. Food Biosci..

[48] Benitez V., Rebollo-Hernanz M., Hernanz S., Chantres S., Aguilera Y., Martin-Cabrejas M.A. (2019). Coffee parchment as a new dietary fiber ingredient: Functional and physiological characterization. Food Biosci..

[49] Ma M.M., Mu T.H. (2016). Effects of extraction methods and particle size distribution on the structural, physicochemical, and functional properties of dietary fiber from deoiled cumin. Food Chem..

